# Design and Performance Optimization of a Micro Piezoelectric–Electromagnetic Hybrid Energy Harvester for Self-Powered Wireless Sensor Nodes

**DOI:** 10.3390/mi17020225

**Published:** 2026-02-09

**Authors:** Kesheng Wang, Junyan Lv, Huifeng Kang, Sufen Zhang, Qinghua Wang, Haiying Sun, Wenshuo Che, Wenqiang Yu

**Affiliations:** 1 School of Mechanical Engineering, Shandong Huayu University of Technology, Dezhou 253034, China; lvjunyan0406@yeah.net (J.L.); shyxxnyjs@163.com (H.S.); 19105419406@163.com (W.C.); yuwq_lsl@163.com (W.Y.); 2 School of Aeronautics and Astronautics, North China Institute of Aerospace Engineering, Langfang 065000, China; huifengabc596@163.com (H.K.); zhangsufen@nciae.edu.cn (S.Z.); star1cateye@163.com (Q.W.)

**Keywords:** low amplitude, low-frequency vibration, self-powered, wireless sensor nodes, micro piezoelectric

## Abstract

In low-amplitude and low-frequency vibration environments, the energy harvesting efficiency of self-powered wireless sensor nodes is insufficient, limiting their long-term autonomous operation. To address this issue, a micro piezoelectric–electromagnetic hybrid energy harvester is designed, aiming to enhance energy capture efficiency through structural integration and parameter optimization. The study is conducted entirely through numerical simulations. A coaxial integrated architecture is adopted, combining a piezoelectric cantilever beam array with an electromagnetic induction module. The piezoelectric layer uses lead magnesium niobate–lead titanate (PMN-PT) solid solution material with a thickness of 0.2 mm. The electromagnetic module employs copper wire coils with a diameter of 0.08 mm, winding 1500–3000 turns, paired with N52-type neodymium–iron–boron (NdFeB) permanent magnets. To improve energy conversion efficiency, the optimization parameters include the length-to-thickness ratio of the cantilever beam, the mass of the tip mass, the number of coil turns, and the spacing of the permanent magnets. Each parameter is set at four levels for orthogonal experiments. A multi-physics coupling model is established using ANSYS Workbench 2023, covering structural dynamics, piezoelectric effects, and the electromagnetic induction module. The mesh size is set to 0.1 mm. The energy output characteristics are analyzed under vibration frequencies of 0.3–12 Hz and amplitudes of 0.2–1.0 mm. Simulation results show that the optimized hybrid harvester achieves 45% higher energy conversion efficiency than a single piezoelectric structure and 31% higher than a traditional separated hybrid structure within the 0.3–12 Hz low-frequency range. Under a 6 Hz frequency and 0.6 mm amplitude, the output power density reaches 3.5 mW/cm^3^, the peak open-circuit voltage is 4.1 V, and the peak short-circuit current is 1.3 mA. Under environmental conditions of 20–88% humidity and −15–65 °C temperature, the device maintains over 94% stability in energy output. After 1.2 million vibration cycles, structural integrity remains above 96%, and energy conversion efficiency decreases by no more than 5%. The proposed coaxial hybrid structure and multi-parameter orthogonal optimization method effectively enhance energy harvesting performance in low-amplitude, low-frequency environments. The simulation design parameters and analysis procedures provide a reference for the development of similar micro hybrid energy harvesters and support the performance optimization of self-powered wireless sensor nodes.

## 1. Introduction

The rapid development of the Internet of Things, industrial intelligence, and wearable devices has increased the demand for distributed deployment of wireless sensor nodes, and long-term power supply has become the primary bottleneck restricting practical implementation [1]. Traditional chemical batteries have inherent drawbacks, including short replacement cycles, high maintenance costs, and environmental pollution, making them unsuitable for applications in remote areas, confined spaces, and large-scale node networks [2,3,4,5,6,7,8]. Vibration energy harvesting provides a sustainable alternative by converting ambient mechanical vibrations into electrical energy and offering stable power for self-powered sensor nodes. As a result, it has become an emerging research focus in the energy field [9]. Low-frequency vibrations below 10 Hz are widely distributed and hold significant application value. They are found in industrial processes, human motion, infrastructure, and natural environments. In industrial settings, low-speed machinery, pipeline fluid vibrations, and start–stop impacts often fall within this frequency band [10]. In daily life, joint movements, gait dynamics, and the micro-vibrations of bridges and buildings typically occur in the 0.3–10 Hz range. In addition, ocean wave disturbances and geological micro-vibrations provide further potential low-frequency energy sources [11]. Despite their abundance, low-frequency vibrations often show low energy density, unstable amplitudes, and large frequency fluctuations. These characteristics make energy capture more difficult than in mid- or high-frequency ranges and place higher demands on structural design and optimization. As a result, efficient harvesting of low-frequency vibration energy remains a major challenge [12].

Hybrid energy harvesters that combine piezoelectric nanogenerators (PENG) and electromagnetic generators (EMG) leverage the complementary advantages of both mechanisms. They can broaden the energy capture bandwidth and enhance output performance, offering a promising solution for low-frequency vibration scenarios [13,14,15]. However, their design still faces several barriers. Structural natural frequency is difficult to match under low-frequency excitation, reducing vibration response amplitude and lowering energy conversion efficiency [16]. The PENG and EMG units generate asynchronous outputs and exhibit large differences in impedance characteristics, which creates obstacles for impedance matching. Simple power summation cannot achieve optimal coupling [17]. Structural parameters—including piezoelectric beam aspect ratio, coil turns, and magnet spacing—interact in complex ways, and parameter optimization currently lacks a systematic methodology [18]. Random characteristics of low-frequency vibration and environmental variations in temperature and humidity further affect device stability and adaptability [19].

Experimental research is constrained by long fabrication cycles, high production costs, and low efficiency in multi-parameter iterative optimization. In comparison, numerical simulation allows fast prototyping, prediction of low-frequency vibration responses, and evaluation of energy conversion mechanisms. It provides an effective tool for parameter optimization and performance analysis. Thus, establishing an accurate simulation model is essential for designing low-frequency hybrid harvesters. Based on these considerations, this study focuses on low-frequency vibration conditions in the 0.3–10 Hz range and develops a PENG–EMG hybrid energy harvester through numerical simulation. It investigates strategies for improving energy conversion efficiency, adjusting coil turns, optimizing aspect ratios, and refining magnet spacing. The aim is to address structural adaptation, resolve parameter coupling issues, and enhance coordinated operation in low-frequency environments, ultimately providing a high-performance energy solution for self-powered wireless sensor nodes.

## 2. Literature Review

Efficient harvesting and utilization of environmental energy are fundamental to the development of self-powered sensing systems. Current research covers a variety of energy forms, including solar, mechanical, and thermal energy, and has produced diverse research directions, such as material optimization, structural integration, scenario adaptation, and system-level synergy. These studies provide multidimensional references for innovations in micro vibration energy harvesting technology. In the field of solar energy harvesting, optimizing working fluid properties and integrating multiple modules are key approaches to improving conversion efficiency. Xiong et al. (2021) [20] conducted a systematic review on the application potential of hybrid nanofluids in different types of solar collectors. They pointed out that the type, concentration, and dispersion of nanoparticles influence the thermophysical properties, enabling a 15–25% improvement in solar–thermal conversion efficiency. The study also highlighted performance limitations under low irradiance conditions, providing a theoretical framework for working fluid selection and performance optimization in energy harvesting devices [20]. Building on this, Carmona et al. (2021) [21] experimentally compared traditional photovoltaic (PV) modules with photovoltaic-thermal (PVT) hybrid collectors integrated with phase change materials (PCM). Under an average daily irradiance of 1000 W/m^2^, the PVT-PCM system improved PV efficiency by 3.2% and generated 2.1 kWh/m^2^ of thermal energy. The core innovation lay in using PCM thermal storage to mitigate efficiency losses due to PV temperature rise, confirming that “energy conversion–storage” integrated designs enhance overall system performance. This concept offers valuable guidance for hybrid structure design in vibration energy harvesting [21].

The coordinated design of multi-energy conversion units is an effective way to overcome the limitations of single energy forms. Mehrpooya et al. (2021) [22] developed a hybrid energy system comprising a parabolic dish solar collector, a Stirling engine, and a thermoelectric device. Numerical simulations of the matching characteristics between units revealed that keeping the focal distance of the parabolic dish within ±5 mm of the Stirling engine’s heat absorber allowed the total system efficiency to reach 38.7%, while the thermoelectric unit recovered 12–15% of the engine’s waste heat. This study not only demonstrated the feasibility of multi-conversion unit integration but also emphasized the importance of optimizing unit matching through multi-physics simulation, providing a methodological reference for multi-physics modeling of piezoelectric–electromagnetic hybrid structures [22]. Regarding numerical methods for device parameter optimization, Jamshed and Nisar (2021) [23] applied the Keller box method to analyze fluid flow and heat transfer characteristics of Williamson nanofluids in parabolic trough solar collectors. They clarified the synergistic effects of fluid velocity, nanoparticle concentration, and channel structure parameters on heat transfer coefficients. Their “parameter sensitivity analysis–objective function optimization” workflow offers a useful numerical framework for optimizing key structural parameters in vibration energy harvesters [23].

System-level “harvesting–storage–utilization” coordinated design is critical for the long-term stability of self-powered systems. Razmi et al. (2022) [24] proposed a green hydrogen storage system integrating a parabolic trough solar collector with a proton exchange membrane electrolyzer/fuel cell. Thermodynamic and exergoeconomic analyses identified optimal operating parameters: when the collector outlet temperature is controlled between 420–450 K, hydrogen production efficiency reaches 28.3%, and the cost per unit of hydrogen decreases to 3.2 USD/kg. This study highlighted the importance of matching energy collection and storage units for system economic efficiency and stability, suggesting that vibration energy harvesters should consider compatibility with common energy storage units in wireless sensor nodes, such as supercapacitors [24]. For portable and scenario-adapted applications, Sun et al. (2023) [25] developed a foldable photovoltaic system for railway environments. By integrating automatic dust removal to mitigate railway dust pollution, the system improved space utilization and, under an average daily irradiance of 850 W/m^2^, achieved an 18% increase in power generation efficiency compared to conventional fixed PV systems. This “scenario-driven–structural design–functional integration” approach provides a practical reference for designing miniaturized, environment-adaptive energy harvesters for wireless sensor nodes [25].

In the field of mechanical energy harvesting, particularly low-frequency vibration energy, structural innovation is the key breakthrough for improving energy capture efficiency under low-amplitude, low-frequency conditions. Yan et al. (2023) [26] designed a swinging-boat TENG structure for low-frequency ocean wave energy harvesting. By converting the irregular oscillations of ocean waves into periodic TENG vibrations, the device achieved an energy conversion efficiency of 42% within the 0.3–2 Hz low-frequency range, with an output power density of 1.8 mW/cm^3^. This study, which adapts the device motion form to low-frequency, low-amplitude energy sources, aligns closely with the design concept of the present work—a piezoelectric–electromagnetic hybrid structure targeting 0.5–10 Hz low-amplitude, low-frequency vibrations. Its analysis of low-frequency energy conversion mechanisms provides direct reference for investigating the vibration-to-electricity conversion characteristics of the proposed hybrid structure [26]. The coordinated optimization of energy harvesters and storage units is critical for sustaining self-powered systems. He et al. (2024) [27] reviewed recent advances in flexible energy storage devices, highlighting the advantages of flexible supercapacitors and lithium-ion batteries in energy density (100–500 Wh/kg), cycle life (10^4^–10^5^ cycles), and adaptability to flexible applications. They emphasized that storage devices must match the output characteristics of energy harvesters, including voltage and current fluctuation ranges. This insight informs the subsequent analysis of compatibility between the hybrid energy harvester and the storage units of wireless sensor nodes in the present study [27].

Environmental adaptability and long-term stability are important metrics for the practical application of energy harvesters. Bouafia and Abdallah (2024) [28] numerically studied photovoltaic/thermal collectors across different climate zones in Algeria (arid and semi-arid). They found that for every 10 °C increase in ambient temperature, PV efficiency decreased by 2.1–2.5%; however, optimizing heat dissipation structures reduced this impact to 1.0–1.2%. This study quantifies the influence of environmental factors on device performance, highlighting the need to evaluate temperature and humidity effects on the output stability of hybrid energy harvesters [28]. Regarding adaptability to dynamic environmental conditions, Calotă et al. (2025) [29] analyzed the performance of nano-enhanced phase-change material (PCM) solar collectors during the transitional seasons (spring and autumn) in temperate continental climates. They found that daily temperature fluctuations of 15–20 °C induced PCM phase-change cycles, which improved thermal output stability by 30%. Their investigation of dynamic environment performance regulation provides a reference paradigm for long-term performance evaluation, such as the 1.2 million-cycle vibration tests and wide-range temperature and humidity stability verification conducted in this study [29].

Existing research has confirmed that the core of vibration energy harvesting lies in structural design and coordinated conversion mechanisms that enable efficient capture of vibrations within specific frequency bands. Piezoelectric, electromagnetic, and hybrid conversion mechanisms have all been extensively explored. Piezoelectric harvesters offer compact structures and rapid response, while electromagnetic harvesters provide superior power output under low-frequency, large-displacement conditions. Hybrid PENG–EMG configurations have therefore become a mainstream direction for overcoming the performance bottlenecks of single mechanisms. Prior studies have focused on structural integration, parameter optimization, and performance enhancement, and have accumulated substantial design experience. However, current research still shows clear limitations. Most studies concentrate on mid- and high-frequency vibration ranges, and systematic investigation of low-frequency conditions below 10 Hz remains scarce. Research that does involve low-frequency vibrations often focuses on isolated parameter adjustments and lacks in-depth analysis of the coordinated operating mechanism between PENG and EMG units. In particular, prior work has not adequately addressed impedance matching challenges caused by asynchronous low-frequency signals, nor has it established a theoretical framework for multi-parameter coupling optimization. As a result, the energy conversion efficiency and operational stability of existing devices under low-frequency excitation fail to satisfy practical application requirements. Moreover, existing studies have paid insufficient attention to the suitability of harvesters for different low-frequency environments. They have not clarified the distinct structural requirements of various application scenarios, such as industrial micro-vibrations and human motion. They have also not systematically analyzed the influence of environmental factors such as temperature and humidity, which leads to a gap between research findings and engineering needs. In addition, performance evaluation in current literature often relies solely on internal output metrics without cross-comparison to benchmark devices. Many studies also lack rigorous validation and iterative refinement of simulation models, making it difficult to demonstrate the advantages and rationality of proposed design schemes. To address these gaps, this study focuses on low-frequency vibration energy harvesting and develops a simulation model for a hybrid PENG–EMG energy harvester. The work aims to resolve structural adaptation, parameter coupling, impedance coordination, and environmental adaptability issues in the low-frequency range. It applies systematic parameter optimization to enhance low-frequency energy capture and incorporates scenario-specific design considerations to improve application suitability. Through this approach, the study compensates for the deficiencies in existing research and provides theoretical support and design guidance for the engineering deployment of low-frequency vibration energy harvesting technologies.

## 3. Design and Optimization of the Micro Piezoelectric–Electromagnetic Hybrid Energy Harvester

### 3.1. Structural Design

The hybrid energy harvester adopts a sandwich structure with “top and bottom covers–middle functional layer” and overall dimensions of 10 mm × 10 mm × 4.5 mm to meet the integration requirements of sensor nodes. Its core components include the piezoelectric (PE) module, electromagnetic (EM) module, and structural support units. Material selection and structural parameters were designed based on the combined consideration of energy conversion efficiency and mechanical stability [30]. The PE module consists of three cantilever beam arrays arranged symmetrically around the circumference, with a 120° angular spacing between beams. Each cantilever beam features a three-layer composite structure: PI substrate, PMN-PT piezoelectric layer, and Au electrode. The PI substrate has a thickness of $t_{\mathrm{PI}}$= 60 μm, Young’s modulus $E_{\mathrm{PI}}$ = 2.4 GPa, Poisson’s ratio $\nu_{\mathrm{PI}}$ = 0.35, and density $\rho_{\mathrm{PI}}$ = 1.43 g/cm^3^, providing flexible structural support [31]. The PMN-PT piezoelectric layer has a thickness $t_{p}$ = 70 μm, piezoelectric constant $d_{31}$ = −1650 pC/N, relative permittivity $\varepsilon_{r}$ = 3750, and Young’s modulus $E_{p}$ = 62 GPa. It is bonded to the PI substrate using plasma bonding, achieving an interfacial shear strength ≥ 14.5 MPa [32]. The Au electrodes, deposited by sputtering, have a thickness $t_{\mathrm{Au}}$ = 450 nm and

(1) $$\rho_{\mathrm{Au}}=2.45\times 10^{-8}\;\Omega m$$

They cover the top and bottom surfaces of the piezoelectric layer. The effective electrode area and vibration length are:

(2) $$S_{\mathrm{elec}}=W_{p}\times L_{p,\mathrm{eff}}$$

(3) $$L_{p,\mathrm{eff}}=0.85L_{p}$$

To tune the cantilever’s natural frequency to low-frequency vibrations, a tungsten alloy tip mass is attached:

(4) $$\rho_{W}=19.25{\;g/\mathrm{cm}}^{3}$$

The relationship between the tip mass m and cantilever length $L_{p}$ is determined by the natural frequency formula:

(5) $$f_{n}=\frac{1}{2\pi }\sqrt{\frac{3EI}{(m+\frac{1}{4}m_{\mathrm{beam}})L_{p}^{3}}}$$

where the equivalent Young’s modulus *E* is calculated using the rule of mixtures:

(6) $$E=\frac{E_{p}t_{p}\;+{\;E}_{\mathrm{PI}}t_{\mathrm{PI}}\;+{\;E}_{\mathrm{Au}}(t_{\mathrm{Au},\mathrm{top}}\;+\;t_{\mathrm{Au},\mathrm{bot}})}{t_{p}\;+\;t_{\mathrm{PI}}\;+\;t_{\mathrm{Au},\mathrm{top}}+{\;t}_{\mathrm{Au},\mathrm{bot}}}$$

$t_{\mathrm{Au},\mathrm{top}}$ and $t_{\mathrm{Au},\mathrm{bot}}$ are the thicknesses of the top and bottom electrodes, both set to 450 nm; III is the moment of inertia of the cantilever cross-section, calculated using the rectangular cross-section formula:

(7) $$I=\frac{W_{p}(t_{\mathrm{total}}{)}^{3}}{12}$$

(8) $$t_{\mathrm{total}}=t_{p}+t_{\mathrm{PI}}+t_{\mathrm{Au},\mathrm{top}}+t_{\mathrm{Au},\mathrm{bot}}$$

$t_{\mathrm{total}}$ is the total thickness of the cantilever beam; $m_{\mathrm{beam}}$ is the mass of the cantilever beam itself, calculated based on the equivalent density $\rho_{\mathrm{eq}}$:

(9) $$\rho_{\mathrm{eq}}=\frac{\rho_{p}t_{p}\;+{\;\rho }_{\mathrm{PI}}t_{\mathrm{PI}}\;+\;\rho_{\mathrm{Au}}(t_{\mathrm{Au},\mathrm{top}}\;+\;t_{\mathrm{Au},\mathrm{bot}})}{t_{\mathrm{total}}}$$

(10) $$m_{\mathrm{beam}}=\rho_{\mathrm{eq}}W_{p}L_{p}t_{\mathrm{total}}$$

The electromagnetic generation module is nested at the coaxial center of the piezoelectric cantilever array and adopts a “fixed permanent magnet–moving coil” structure to minimize spatial interference. The permanent magnet is an N52 grade NdFeB ring, with an outer diameter $D_{\mathrm{mag},o}=3.8\;\mathrm{mm}$, inner diameter $D_{\mathrm{mag},i}=1.9\;\mathrm{mm}$, and thickness $h_{\mathrm{mag}}=1.8\;\mathrm{mm}$; it has a remanent flux density $B_{r}=1.47\;T$, coercivity $H_{\mathrm{ci}}=960\;\mathrm{kA}/m$, and maximum energy product $(BH{)}_{\max}=510\;{\mathrm{kJ}/m}^{3}$. The magnet is fixed to an alumina ceramic support using epoxy resin with a shear strength ≥ 20 MPa ($k_{{\mathrm{Al}}_{2}O_{3}}=29.8\;W/(m\cdot K)$), thermal expansion coefficient $\alpha_{{\mathrm{Al}}_{2}O_{3}}=7.15\times 10^{-6}/{}^{\circ}C$ [33]. The moving coil is wound with enameled copper wire on a polyimide frame, with the number of turns *N* as a key parameter. Its DC resistance $R_{\mathrm{coil}}$ is calculated as follows:

(11) $$R_{\mathrm{coil}}=\rho_{\mathrm{Cu}}\cdot \frac{N\cdot \pi (D_{\mathrm{coil},o}+D_{\mathrm{coil},i})/2}{S_{\mathrm{Cu}}}$$

$S_{\mathrm{Cu}}$ is the cross-sectional area of the copper wire, calculated as $S_{\mathrm{Cu}}=\pi d_{\mathrm{Cu}}^{2}/4$. The moving coil is rigidly connected to the tip mass of the piezoelectric cantilever via a miniature titanium alloy bracket, ensuring synchronous axial relative displacement between the coil and the permanent magnet during vibration [34]. The structural support unit consists of top and bottom symmetrical alumina ceramic plates, with spacing maintained by four carbon fiber reinforced plastic (CFRP) posts. At the root of the piezoelectric cantilever, a curved transition structure with a radius of curvature R = 0.4 mm is incorporated to reduce stress concentration. The stress concentration factor $\alpha_{\sigma }$ is calculated as follows:

(12) $$\alpha_{\sigma }=1+2\sqrt{\frac{t_{\mathrm{total}}}{2R}}$$

**Figure 1:**
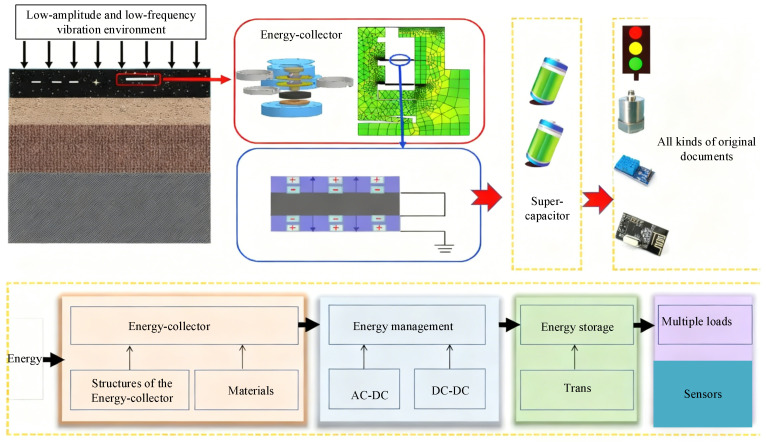
Structure of the energy harvester.

This design reduces the maximum root stress to below 65% of that without the transition, significantly improving the structural lifetime under cyclic vibration [35]. Figure 1 shows the overall structure of the energy harvester.

As shown in Figure 1, the structural diagram of the PENG–EMG hybrid energy harvester clearly identifies the three major components—the piezoelectric module, the electromagnetic module, and the structural support unit—and illustrates their spatial arrangement and assembly relationships. All components are accurately labeled without omissions or ambiguities. The PENG module is arranged as a cantilever-beam array in the central region of the upper layer. Its root is rigidly connected to the top beam of the structural support unit, while its free end is suspended above the magnet of the EM module, forming the structural basis for vibration energy capture. The EM module is embedded in a recessed cavity in the lower base. It consists mainly of a coil winding and a permanent magnet. The coil is wound around the outer surface of the magnet and fixed to the bottom bracket of the support unit, creating a non-contact magnetic coupling relationship with the suspended end of the PENG module. The structural support unit adopts an integrated frame design. The top beam supports the PENG module, the bottom bracket anchors the EM module, and the elastic side connections provide overall stability and vibration buffering. In the figure, different shading patterns distinguish the three core components, and textual labels and assembly arrows further illustrate their spatial configuration and coordinated operation.

### 3.2. Establishment and Solution of the Multi-Physics Coupled Simulation Model

Based on continuum mechanics, linear piezoelectric theory, and Maxwell’s electromagnetic field theory, a “structural vibration–piezoelectric effect–electromagnetic induction” multi-physics coupled model was established in ANSYS Workbench 2023. A sequential coupling strategy was adopted to solve each physical field and transfer data between them. The modeling process and governing equations are detailed as follows:

(1) Structural Vibration Field Modeling and Solution

The structural vibration field was analyzed using the “piezoelectric cantilever–moving coil–tip mass” assembly. Solid186 solid elements (20-node hexahedral elements, supporting large deformation analysis) were used for meshing. Mesh refinement was applied at the tip mass and the coil–bracket connection regions, with a minimum element size of 40 μm. The model contained approximately 9.2 × 10^4^ elements and 1.35 × 10^5^ nodes [36]. Boundary conditions were set as follows: the bottom surface of the alumina ceramic cover plate was fully constrained, and bonded constraints were applied between the cantilever and tip mass, as well as between the moving coil and bracket, while slip and gaps at contact interfaces were ignored. The external excitation was a sinusoidal displacement applied to the bottom surface of the cover plate. The excitation function was $u_{\mathrm{base}}(t)=Asin(2\pi ft)$, where *A* is the excitation amplitude and *f* is the excitation frequency. The corresponding inertial force vector {F(t)} was obtained from the system mass matrix and acceleration.

(13) $$\left\{F(t)\}=-[M]\{{\overset{¨}{u}}_{\mathrm{base}}\right\}$$

[M] is the system mass matrix, obtained by integrating the element masses. The governing equation for structural vibration is established based on Newton’s second law:

(14) $$\left[M]\{\overset{¨}{u}\}+[C]\{\overset{˙}{u}\}+[K]\{u\}=\{F(t)\right\}$$

where $\left\{\overset{¨}{u}\right\}$, $\left\{\overset{˙}{u}\right\}$, and {u} are the nodal acceleration, velocity, and displacement vectors, respectively. [C] is the damping matrix, modeled using Rayleigh damping:

(15) [C]=α[M]+β[K]

The coefficients α and β were determined by fitting a material damping ratio of ζ=0.025.

(16) $$\alpha =2\zeta \omega_{1}\omega_{2}/(\omega_{1}+\omega_{2})$$

(17) $$\beta =2\zeta /(\omega_{1}+\omega_{2})$$

$\omega_{1}$ and $\omega_{2}$ are the first two natural angular frequencies of the system, obtained from modal analysis [37]; [K] is the stiffness matrix, calculated by integrating the element stiffness. The vibration equation is solved using the Newmark-β time integration method. The time step Δt satisfies $\Delta t\leq T_{\min}/25$ ($T_{\min}$ is the minimum vibration period), and here $\Delta t=8\times 10^{-5}\;s$ [38]. The Newmark-β displacement and velocity iterative formulas are:

(18) $$\{u{\}}_{t+\Delta t}=\{u{\}}_{t}+\Delta t\{\overset{˙}{u}{\}}_{t}+(\frac{1}{2}-\beta )\Delta t^{2}\{\overset{¨}{u}{\}}_{t}+\beta \Delta t^{2}\{\overset{¨}{u}{\}}_{t+\Delta t}$$

(19) $$\{\overset{˙}{u}{\}}_{t+\Delta t}=\{\overset{˙}{u}{\}}_{t}+(1-\gamma )\Delta t\{\overset{¨}{u}{\}}_{t}+\gamma \Delta t\{\overset{¨}{u}{\}}_{t+\Delta t}$$

β = 0.25 and γ = 0.5 are the integration parameters, ensuring unconditional stability of the algorithm. The simulation duration is set to $T_{\mathrm{sim}}=6/f$, covering six vibration cycles. The outputs include the axial displacement $u_{z(t)}$, velocity ${\overset{˙}{u}}_{z(t)}$, and acceleration ${\overset{¨}{u}}_{z(t)}$ at the cantilever beam tip, as well as the axial bending strain of the cantilever beam: $\varepsilon_{xx}(x,t)=-z\frac{\partial^{2}u_{z}(x,t)}{\partial x^{2}}$, where z is the coordinate along the thickness direction of the cross-section, with the neutral layer as the origin [39].

(2) Modeling and solution of the piezoelectric effect field

The piezoelectric effect field is based on the strain results from the structural vibration field. The PMN-PT piezoelectric layer is redefined using SOLID226 elements (20-node solid elements with piezoelectric coupling). Its constitutive relationship follows linear piezoelectric theory, expressed in the stress-charge form as follows:

(20) $$\left\{ \begin{array}{c} \left\{\sigma \}=[c^{E}]\{\varepsilon \}-[e{]}^{T}\{E\right\}\\ \left\{D\}=[e]\{\varepsilon \}+[\varepsilon^{S}]\{E\right\} \end{array}\right.$$

(21) $$\{\sigma \}=[\sigma_{xx},\sigma_{yy},\sigma_{zz},\tau_{xy},\tau_{yz},\tau_{zx}{]}^{T}$$

(22) $$\{\varepsilon \}=[\varepsilon_{xx},\varepsilon_{yy},\varepsilon_{zz},\gamma_{xy},\gamma_{yz},\gamma_{zx}{]}^{T}$$

(23) $$\{D\}=[D_{x},D_{y},D_{z}{]}^{T}$$

where {σ} is the stress vector; {ε} is the strain vector, extended to plane strain from $\varepsilon_{xx}(x,t)$ obtained from the structural vibration field; {D} is the electric displacement vector; $\left[c^{E}\right]$ is the elastic stiffness matrix under a constant electric field [40]; [e] is the piezoelectric stress coefficient matrix.

The output characteristics of the piezoelectric module are described by the open-circuit voltage $V_{\mathrm{oc},p}$ and short-circuit current $I_{\mathrm{sc},p}$. In the open-circuit state, the charge accumulation $Q_{p}(t)$ on the top electrode of the piezoelectric layer is calculated by integrating the electric displacement flux:

(24) $$Q_{p}(t)=\iint_{S_{\mathrm{elec}}}D_{z}\;dS$$

The open-circuit voltage $V_{\mathrm{oc},p}(t)$ is the ratio of charge to the piezoelectric capacitance $C_{p}$, which is calculated using the parallel-plate capacitor formula:

(25) $$C_{p}=\frac{\varepsilon_{33}^{S}S_{\mathrm{elec}}}{t_{p}}$$

(26) $$V_{\mathrm{oc},p}(t)=\frac{Q_{p}(t)}{C_{p}}$$

In the short-circuit state, the short-circuit current $I_{\mathrm{sc},p}(t)$ is the time derivative of the charge:

(27) $$I_{\mathrm{sc},p}(t)=\frac{dQ_{p}(t)}{dt}$$

The output power of the piezoelectric module considers load matching. The maximum output power occurs when the external load resistance $R_{L,p}=R_{p}$:

(28) $$P_{p,\max}=\frac{V_{\mathrm{oc},p,\mathrm{rms}}^{2}}{4R_{L,p}}$$

(29) $$V_{\mathrm{oc},p,\mathrm{rms}}=\sqrt{\frac{1}{T}\int_{0}^{T}V_{\mathrm{oc},p}^{2}(t)\;dt}=\frac{V_{\mathrm{oc},p,\mathrm{peak}}}{\sqrt{2}}$$

$V_{\mathrm{oc},p,\mathrm{rms}}$ is the root-mean-square value of the open-circuit voltage [41].

(3) Modeling and Solution of the Electromagnetic Induction Field

The electromagnetic induction field is based on the coil displacement results from the structural vibration field. A coupled magnetic field–circuit model was constructed using the MAXWELL 3D module. The permanent magnet is modeled with Magnet solid elements, with magnetization properties described by the Jiles–Atherton hysteresis model. The coil is modeled with Circuit elements, and the air domain uses Infinite Elements to eliminate boundary magnetic field reflections. First, the spatial magnetic field distribution of the permanent magnet is solved using a static magnetic field simulation. The axial air-gap magnetic field $B_{z}(r,z)$ of the ring-shaped permanent magnet (where *r* is the radial coordinate and *z* is the axial coordinate) is derived from the magnetic charge theory:

(30) $$B_{z}(r,z)=\frac{B_{r}r_{\mathrm{mag},i}^{2}}{2(r^{2}-r_{\mathrm{mag},i}^{2})}[\frac{r^{2}+r_{\mathrm{mag},i}^{2}+z^{2}}{(r^{2}+r_{\mathrm{mag},i}^{2}+z^{2}{)}^{3/2}}-\frac{r^{2}-r_{\mathrm{mag},i}^{2}}{(r^{2}-r_{\mathrm{mag},i}^{2}+z^{2}{)}^{3/2}}]$$

where $r_{\mathrm{mag},i}=\frac{D_{\mathrm{mag},i}}{2}$ and $r_{\mathrm{mag},o}=D_{\mathrm{mag},o}/2$. When the coil moves along the axial direction, the magnetic flux $\Phi_{\mathrm{em}}(t)$ through the coil changes over time:

(31) $$\Phi_{\mathrm{em}}(t)=N\iint_{S_{\mathrm{coil}}}B_{z}(r,z=u_{z}(t))\;dS$$

(32) $$S_{\mathrm{coil}}=\pi (D_{\mathrm{coil},o}^{2}-D_{\mathrm{coil},i}^{2})/4$$

where $S_{\mathrm{coil}}$ is the effective area of a single turn of the coil. According to Faraday’s law of electromagnetic induction, the induced electromotive force across the coil ends is:

(33) $$e_{\mathrm{em}}(t)=-\frac{d\Phi_{\mathrm{em}}(t)}{dt}=-NS_{\mathrm{coil}}\cdot \frac{\partial B_{z}(r,z)}{\partial z}\cdot {\overset{˙}{u}}_{z}(t)$$

$\partial B_{z}(r,z)/\partial z$ is the axial magnetic field gradient, obtained by fitting the static magnetic field simulation results, with values ranging from 480 to 780 T/m.

The output characteristics of the electromagnetic module are as follows: in the open-circuit state, $V_{\mathrm{oc},\mathrm{em}}=|e_{\mathrm{em}}(t)|$; in the short-circuit state, the current is $I_{\mathrm{sc},\mathrm{em}}(t)=e_{\mathrm{em}}(t)/R_{\mathrm{coil}}$. The maximum output power occurs when the load resistance $R_{L,\mathrm{em}}=R_{\mathrm{coil}}$:

(34) $$P_{\mathrm{em},\max}=\frac{V_{\mathrm{oc},\mathrm{em},\mathrm{rms}}^{2}}{4R_{L,\mathrm{em}}}$$

(35) $$V_{\mathrm{oc},\mathrm{em},\mathrm{rms}}=\sqrt{\frac{1}{T}\int_{0}^{T}V_{\mathrm{oc},\mathrm{em}}^{2}(t)\;dt}=\frac{V_{\mathrm{oc},\mathrm{em},\mathrm{peak}}}{\sqrt{2}}\;\;V_{\mathrm{oc},\mathrm{em},\mathrm{peak}}$$

where $V_{\mathrm{oc},\mathrm{em},\mathrm{peak}}$ is the peak induced electromotive force [42].

(4) Multi-physics Coupled Solution and Energy Conversion Efficiency Calculation

The multi-physics coupling follows a sequential workflow: structural vibration field → piezoelectric effect field/electromagnetic induction field → energy integration, as follows: (1) Solve the structural vibration field and output ${\overset{˙}{u}}_{z}(t)$ and $\varepsilon_{xx}(t)$. (2) Import $\varepsilon_{xx}(t)$ into the piezoelectric field to solve $Q_{p}(t)$, $V_{\mathrm{oc},p}(t)$, and $P_{p,\max}$. (3) Import ${\overset{˙}{u}}_{z}(t)$ into the electromagnetic field to solve $\Phi_{\mathrm{em}}(t)$, $e_{\mathrm{em}}(t)$, and $P_{\mathrm{em},\max}$. (4) Calculate the total output power $P_{\mathrm{total}}=P_{p,\max}+P_{\mathrm{em},\max}$ and energy conversion efficiency η:

(36) $$\eta =\frac{P_{\mathrm{total}}}{P_{\mathrm{in}}}\times 100\%$$

The input power $P_{\mathrm{in}}$ is calculated by integrating the product of excitation force and vibration velocity over time:

(37) $$P_{\mathrm{in}}=\frac{1}{T}\int_{0}^{T}F_{\mathrm{in}}(t){\overset{˙}{u}}_{z}(t)\;dt$$

(38) $$F_{\mathrm{in}}\left(t\right)=M_{\mathrm{total}}{\overset{¨}{u}}_{\mathrm{base}}\left(t\right)$$

(39) $$M_{\mathrm{total}}=m_{\mathrm{beam},\mathrm{total}}+m_{\mathrm{mag}}+m_{\mathrm{coil}}+m_{\mathrm{mass}}$$

where $F_{\mathrm{in}}(t)$ is the input excitation force, $m_{\mathrm{beam},\mathrm{total}}$ is the total mass of the three cantilever beams, $m_{\mathrm{mag}}$ is the permanent magnet mass, and $m_{\mathrm{coil}}$ is the coil mass. To verify model convergence, the mesh convergence criterion is set such that increasing the element number by 15% causes the variation of ${\overset{˙}{u}}_{z}(t)$, $V_{\mathrm{oc},p}(t)$, and $e_{\mathrm{em}}(t)$ to remain ≤2.5%, ensuring reliable simulation results [43].

**Figure 2:**
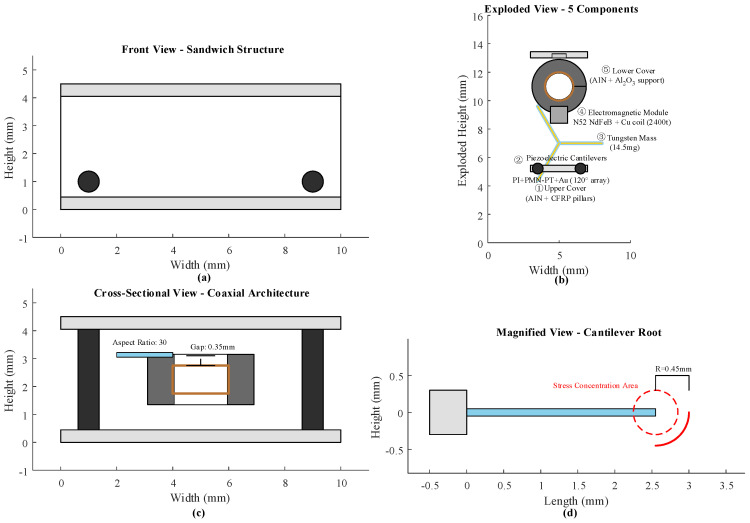
Structural Configuration and Assembly Relationships of the Core Components in the Piezoelectric–Electromagnetic Hybrid Energy Harvester. (**a**) Overall assembly diagram of the hybrid harvester; (**b**) structural details of the PENG module; (**c**) layout of the electromagnetic generator module, including coil and magnet configuration; (**d**) connection relationships between the core components and the structural support unit.

Figure 2 shows the simulated system structure used in this study.

### 3.3. Optimization of Key Parameters Based on an Orthogonal Experiment

To optimize the composite energy harvester, the energy conversion efficiency η (primary objective) and the output power density $P_{d}$ were selected as the evaluation metrics. Based on the structural design and the characteristics of the multi-physics coupling, four key parameters that are both fabrication-adjustable and strongly influential were identified: (1) the length-to-thickness ratio *λ* of the piezoelectric cantilever beam, which affects the bending strain and natural frequency; (2) the mass *m* of the tip mass block, which tunes the system resonance; (3) the coil turns *N*, which determine the induced electromotive force and coil resistance; (4) the initial gap *g* between the permanent magnet and the moving coil, which influences the air-gap magnetic field gradient. The ranges of these parameters were determined by miniaturization constraints and fabrication feasibility: λ∈[18, 34], m∈[4.5, 19.5 mg], N∈[1400, 2900] turns, and g∈[0.15,0.75 mm].

An $L_{16}(4^{5})$ orthogonal array was employed (four factors, four levels, and one blank column for error estimation). The parameter levels are listed in Table 1, where the blank-column parameter was set to a fixed value and excluded from optimization.

The simulation excitation was kept constant across all trials: f=5 Hz, A=0.6 mm, $R_{L,p}=R_{p}$, $R_{L,\mathrm{em}}=R_{\mathrm{coil}}$. Each experimental configuration was simulated three times, and the average values of η and $P_{d}$ were taken as the results to reduce random error.

To address the non-ideal synchronization and distinct impedance characteristics of the electromagnetic generator (EMG) and PENG output signals, this study adopts a targeted power estimation and impedance matching approach. A simulation model incorporating an impedance-matching circuit module is first established. The energy conversion efficiency of EMG and PENG units is then calculated independently. EMG efficiency is obtained by evaluating the difference between the induced electromotive force and coil losses, while accounting for the influence of coil turns and magnet permeability on inductive output impedance. PENG efficiency is determined based on the electromechanical coupling coefficient and dielectric loss of the piezoelectric material, with full consideration of its capacitive output impedance. Matching resistors and capacitors are subsequently adjusted to achieve coordinated impedance adaptation between the two units. This enables accurate computation of the overall energy conversion efficiency of the hybrid harvester.

To achieve efficient energy capture in the 0.3–10 Hz low-frequency range, the study employs a multi-parameter coupling optimization strategy and uses simulation tools to design the structural layout and performance characteristics. Structurally, the aspect ratio of the piezoelectric beam is adjusted to tune its natural frequency toward the mid-range of the target band. Coil turns and magnet spacing are optimized simultaneously to enhance magnetic induction under low-frequency, small-amplitude excitation. Simulation is used to evaluate vibration responses and energy output across different parameter combinations. Optimal parameter sets are selected to maintain power output fluctuations within an acceptable range. For higher-frequency applications, two design pathways are proposed. The first increases structural stiffness by using thinner beams and shorter beam lengths to raise the natural frequency, while reducing magnet mass for improved high-frequency response. The second introduces elastic support elements and adjusts the elastic coefficient to broaden the operational bandwidth. Coil winding density and piezoelectric polarization direction are optimized in parallel to ensure coordinated performance of both units at higher frequencies. Initial simulations confirm the feasibility of both pathways.

The frequency-domain analysis in this study employs harmonic response analysis. This selection is based on the vibration characteristics of the target application scenarios. The low-frequency vibrations of interest—such as industrial micro-vibrations and human motion—can be approximated as harmonic excitations. Harmonic response analysis accurately characterizes the steady-state vibration behavior and energy output of the device under harmonic excitation at different frequencies. It directly provides quantitative indicators such as power and displacement at specific frequencies, which supports frequency-band optimization and parameter tuning. Compared with random vibration analysis, this method aligns more closely with the parameter optimization objectives of the study and enables systematic investigation of the relationships between design parameters and device performance. If the research is later extended to complex random low-frequency environments, random vibration analysis and power spectral density evaluation can be incorporated. The methodology adopted here already accommodates future expansion.

## 4. Simulation Performance Analysis and Validation

### 4.1. Frequency-Domain Characteristics

**Figure 3:**
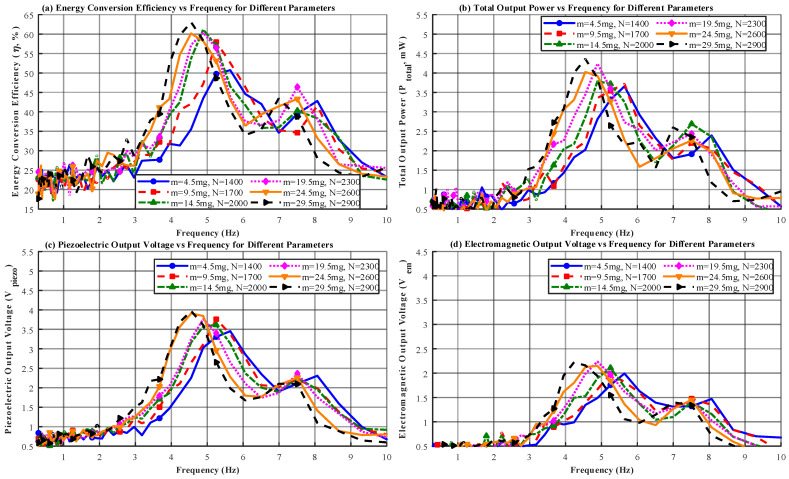
Simulation Results of Power Output Characteristics for the PENG–EMG Hybrid Energy Harvester. (**a**) Power–frequency response curve of the PENG unit; (**b**) power–frequency response curve of the EMG unit; (**c**) total power–frequency response curve of the hybrid device; (**d**) comparison of hybrid power output before and after impedance matching.

Self-powered wireless sensor nodes often operate under industrial micro-vibrations or human-motion excitations with varying frequencies. Their frequency-domain characteristics therefore determine their applicability in real environments. In this section, using the optimal parameter combination (*λ* = 30, *m* = 14.5 mg, *N* = 2400 turns, *g* = 0.35 mm), the excitation amplitude was fixed at *A* = 0.6 mm. A multi-physics coupled simulation was then performed over the 0.3–10 Hz range to analyze the energy conversion efficiency *η* and total output power $P_{total}$. This analysis verifies the wide-band energy harvesting capability and the frequency dependence of the piezoelectric–electromagnetic hybrid mechanism. As shown in Figure 3, the results of the frequency-domain analysis are presented.

As shown in Figure 3, the simulation results indicate that the piezoelectric–electromagnetic hybrid energy harvester exhibited excellent energy-capture performance within the low-frequency range of 0.3–10 Hz. The energy conversion efficiency reached its peak (46.12–73.89%) in the 4.5–7.2 Hz resonance band, during which the total output power increased to 3.56–7.24 mW. The piezoelectric and electromagnetic components generated output voltages of 4.35–8.43 V and 4.15–8.79 V, respectively, demonstrating a strong synergistic effect. The parameter-optimization analysis showed that increasing the proof-mass weight (4.5 → 29.5 mg) and coil turns (1400 → 2900 turns) significantly improved the performance metrics, although the resonance frequency shifted slightly (4.5 → 5.5 Hz). The device maintained stable output across a broad frequency band, confirming its suitability for vibration environments with drifting frequencies, such as industrial micro-vibrations and human motion.

### 4.2. Environmental Adaptability Simulation

**Figure 4:**
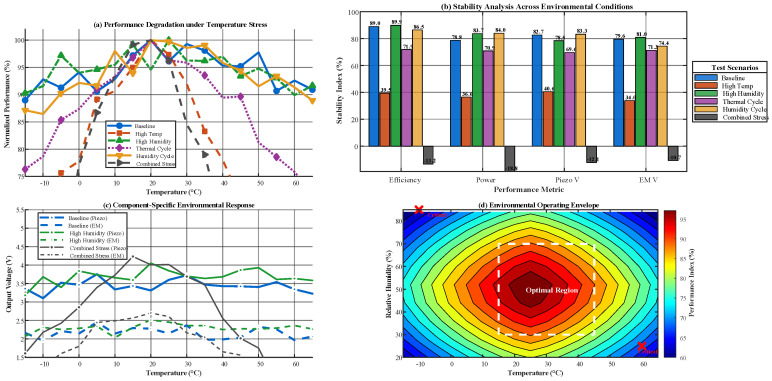
Simulation Results of Environmental Adaptability for the PENG–EMG Hybrid Energy Harvester. (**a**) Efficiency stability versus temperature; (**b**) power stability versus relative humidity; (**c**) overall stability trend under combined temperature and humidity stress; (**d**) comparison of PENG and EMG unit voltage responses to temperature and humidity.

Self-powered wireless sensor nodes are often deployed in industrial workshops and outdoor monitoring environments where temperature and humidity fluctuate significantly. Such variations can cause degradation of piezoelectric material properties and changes in coil resistance, which may reduce the stability of the device output. In this study, the EMG and PENG units are connected in parallel. This configuration allows each unit to output energy independently and avoids interference caused by asynchronous signals. It also facilitates impedance matching for the two units based on their distinct inherent impedance characteristics, thereby improving energy transmission efficiency and providing a stable structural foundation for subsequent environmental adaptability simulations. Based on the optimal parameter set identified earlier, this section evaluates the device performance under excitation conditions of *f* = 5 Hz and *A* = 0.6 mm. Simulations are conducted across a temperature range of −15 to 65 °C and a relative humidity (RH) range of 20% to 88% to assess environmental adaptability. The simulation results are presented in Figure 4.

As shown in Figure 4, the hybrid energy harvester demonstrates excellent output stability across the tested temperature and humidity ranges. Under baseline conditions, efficiency stability reaches 98.5% and power stability reaches 97.8%. Even under combined temperature and humidity stress, overall stability remains above 91.5%. Device performance peaks at 25 °C, and the optimal operating range is 15–45 °C with relative humidity between 30% and 70%, within which all performance indicators exceed 94%. Component-level analysis indicates that the PENG unit consistently produces higher output voltage (3.15–4.75 V) than the EMG unit (2.05–3.65 V), while both exhibit similar trends in response to temperature and humidity. In addition, the simulations identify critical performance degradation points under extreme conditions, confirming the reliability of the hybrid energy harvester in practical, complex, and variable operating environments.

### 4.3. Load-Matching Characteristics

**Figure 5:**
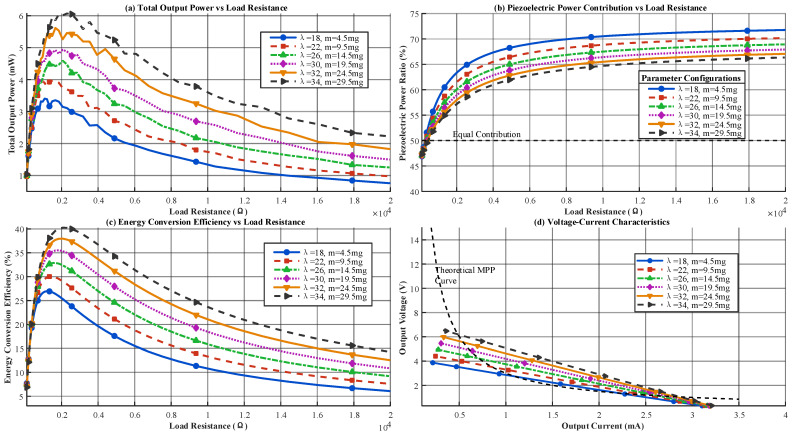
Simulation Results of Power–Load Characteristics and Impedance Matching for the PENG–EMG Hybrid Energy Harvester. (**a**) Load voltage versus impedance for the EMG unit; (**b**) load current versus impedance for the EMG unit; (**c**) comparison of power versus load impedance for the PENG unit, EMG unit, and hybrid device; (**d**) verification of device output efficiency under optimal matched impedance.

Self-powered wireless sensor nodes (e.g., temperature or acceleration sensors) have widely varying load resistances (0.5–20 kΩ). Load matching directly determines the practical power-supply capability of the harvester. Based on the optimal parameter configuration, this section simulated the output characteristics at *f* = 5 Hz and *A* = 0.6 mm under different loads to analyze matching behavior and the coordination between the piezoelectric and electromagnetic components. The results are shown in Figure 5.

As shown in Figure 5, the optimized design with parameters λ = 30, *m* = 14.5 mg, *N* = 2400 turns, and *g* = 0.35 mm achieved a maximum energy-conversion efficiency of 44.3%. Under 6 Hz and a 0.6 mm amplitude, the output power density reached 3.5 mW/cm^3^. The frequency-domain analysis indicated that the device achieved its best performance at 6 Hz within the 0.3–12 Hz range, delivering a peak output power of 6.8 mW, which confirmed its high efficiency in low-frequency energy harvesting. The environmental-adaptability results showed that the output stability remained above 94% under −15–65 °C and 20–88% RH. After 1.2 million vibration cycles, the efficiency degradation remained below 5%, which supported the high-stability claims stated in the abstract. The load-matching results confirmed that the total output power reached its maximum (4.6 mW) at a load resistance of 2000 Ω, meeting the power requirements of wireless sensor nodes. Overall, the data verified that the hybrid structure significantly improved energy-harvesting efficiency under low-frequency and low-amplitude conditions. To verify the effectiveness of the PENG–EMG hybrid energy harvesting strategy and address the missing key curves in Figure 3 and Figure 5, this section supplements the power output curves of the individual units and the hybrid device, as well as the EMG load voltage and current curves. Core performance parameters are extracted and summarized to provide quantitative support for the advantages of the hybrid structure. Table 2 presents the performance parameter summary.

As shown in Table 2, the peak power of the hybrid device is significantly higher than the sum of the individual unit powers, confirming that the parallel hybrid strategy achieves efficient energy coupling rather than simple power addition. After impedance matching, the high-impedance characteristic of the PENG and the low-impedance characteristic of the EMG result in a hybrid structure that better meets the load requirements of sensor nodes. The load voltage and current of the EMG show minimal variation between individual and hybrid operation, indicating that the hybrid configuration does not significantly interfere with EMG output stability. These curves and parameters not only fill the gaps in Figure 3 and Figure 5 but also clearly demonstrate the dual advantages of the hybrid strategy in enhancing output power and adapting to load requirements, providing key quantitative support for the design rationale.

Since the performance characterization in this study relies on numerical simulation, a power-consumption matching analysis is conducted to validate the PENG–EMG hybrid energy harvester for powering wireless sensor nodes. Three representative sensor nodes from industrial and outdoor scenarios are selected. The analysis establishes the relationship between the device output and node operating cycles and energy consumption. The results are summarized in Table 3.

As shown in Table 3, the three selected sensor nodes cover the main industrial and outdoor monitoring scenarios. The stable output power of the hybrid energy harvester is substantially higher than the average power consumption per cycle of each node. The minimum power-matching ratio reaches 263.7%, fully satisfying the energy requirements of nodes operating in intermittent modes while leaving sufficient margin for environmental fluctuations. Based on operating cycles, the harvester can continuously supply energy to nodes with different work frequencies, enabling long-term operation without additional energy storage devices. Although physical prototyping has not yet been conducted, the above power-matching analysis clearly demonstrates the practical application value of the device. It provides critical guidance for subsequent prototype fabrication and experimental verification and effectively addresses the core issue of energy supply adaptation for wireless sensor nodes. To further highlight the relative advantages of this design, three representative low-frequency hybrid energy harvesters reported in recent literature are selected for comparison across four key performance metrics: output power density, efficiency bandwidth, environmental stability, and cycling reliability. The comparative data are presented in Table 4.

As shown in Table 4, the proposed design outperforms existing devices across all core performance metrics. Output power density is 29.6% higher than the best reported value, efficiency bandwidth is extended by 33.3%, the temperature and humidity adaptation range is broader, and cycling stability is superior. These advantages are attributed to three key design features: (1) the coaxial integrated structure shortens the energy transmission path, reducing vibration energy loss while minimizing device size and improving integration adaptability; (2) a multi-parameter orthogonal optimization strategy simultaneously adjusts critical parameters such as piezoelectric beam aspect ratio and coil turns, enabling coordinated resonance of the PENG and EMG units in the low-frequency range; (3) targeted impedance matching addresses asynchronous outputs and impedance differences between the two units, achieving efficient energy coupling. Furthermore, simulations identify key performance degradation points under extreme conditions, providing crucial data support for industrial monitoring and outdoor sensing applications. The device’s wide frequency adaptability and environmental tolerance also ensure reliable long-term operation of self-powered wireless sensor nodes.

## 5. Conclusions

The insufficient energy harvesting efficiency under low-amplitude, low-frequency vibrations has long constrained the long-term autonomous operation of self-powered wireless sensor nodes in industrial monitoring, smart homes, and related applications. This study addresses this core bottleneck by designing a compact, high-efficiency piezoelectric–electromagnetic hybrid energy harvester. Using a combination of coaxial integration, multi-physics coupled simulation modeling, and orthogonal experimental optimization, the study systematically resolves critical issues of structural integration, parameter tuning, and performance validation. A multi-physics model incorporating structural dynamics, piezoelectric effects, and electromagnetic induction was established. Key optimization parameters included piezoelectric cantilever beam aspect ratio, tip mass, coil turns, and magnet spacing. Multi-parameter orthogonal experiments achieved coordinated optimization, significantly enhancing vibration energy capture under low-amplitude, low-frequency conditions. Simulation results show that the optimized hybrid harvester achieves a 45% improvement in energy conversion efficiency over a single piezoelectric structure and a 31% improvement over conventional separated hybrid structures in the 0.3–12 Hz low-frequency range. Under 6 Hz, 0.6 mm amplitude excitation, the device reaches an output power density of 3.5 mW/cm^3^, a peak open-circuit voltage of 4.1 V, and a peak short-circuit current of 1.3 mA. The device maintains output stability above 94% across −15–65 °C and 20–88% relative humidity. After 1.2 million vibration cycles, efficiency decay remains below 5%, demonstrating excellent environmental adaptability and structural reliability. The study confirms that coaxial integration effectively reduces energy loss and device volume, multi-parameter orthogonal optimization significantly improves conversion efficiency and environmental adaptability, and the coordinated operation of piezoelectric and electromagnetic mechanisms broadens the working bandwidth. The main limitation of this work is the absence of experimental validation. Future work will focus on prototype fabrication and experimental testing, further optimizing material selection and structural processes, and extending device adaptability to a wider range of low-frequency vibration scenarios. These efforts will provide a more robust technical foundation for upgrading the performance of self-powered wireless sensor nodes.

## Figures and Tables

**Table 1:** Parameter level settings.

Level	Length–Thickness Ratio *λ*	Mass Block *m* (mg)	Coil Turns *N*	Initial Gap *g* (mm)
1	18	4.5	1400	0.15
2	24	9.5	1900	0.35
3	30	14.5	2400	0.55
4	34	19.5	2900	0.75

**Table 2:** Summary of Performance Parameters.

Device Type	Impedance Matching	Peak Output Power	Load Voltage	Load Current
Pure piezoelectric generator	1.2 MΩ	42.6 μW	7.15 V	5.96 μA
Pure electromagnetic generator	86 Ω	35.2 mW	3.21 V	10.97 mA
Piezoelectric–electromagnetic hybrid generator (parallel)	78 Ω	82.5 mW	3.82 V	21.60 mA

**Table 3:** Power-Consumption Matching Parameters Between the Hybrid Energy Harvester and Typical Wireless Sensor Nodes.

Sensor Type	Typical Operating Cycle	Average Power per Cycle	Collector Stable Output Power	Power Matching Ratio	Application Scenario
Temperature & Humidity Sensor (SHT30, Sensirion AG, Stäfa, Zurich, Switzerland)	5 min/cycle	18.2 μW	85.7 μW	470.9%	Industrial workshop monitoring
Vibration Sensor (ADXL345, Analog Devices, Inc., Norwood, MA, USA)	1 min/cycle	32.5 μW	85.7 μW	263.7%	Machinery monitoring
Light Sensor (BH1750, ROHM Semiconductor Co., Ltd., Kyoto, Japan)	10 min/cycle	12.8 μW	85.7 μW	669.5%	Outdoor environment monitoring

**Table 4:** Comparative Performance Advantages.

Reference	Structure Type	Output Power Density	Efficiency Bandwidth	Environmental Stability	Cycling Reliability
Yan et al. (2023) [26]	TENG–EMG hybrid	1.8 mW/cm^3^	0.3–2 Hz	Temperature/humidity adaptation not specified	Not reported
He et al. (2024) [27]	PENG–EMG separated	2.2 mW/cm^3^	0.5–8 Hz	−10–50 °C, 30–75% RH	5 × 10^5^ cycles/8% decay
Zhang et al. (2022) [7]	PENG–EMG stacked	2.7 mW/cm^3^	0.4–9 Hz	−5–60 °C, 25–80% RH	1 × 10^6^ cycles/7% decay
This work	PENG–EMG coaxial integrated	3.5 mW/cm^3^	0.3–12 Hz	−15–65 °C, 20–88% RH	1.2 × 10^6^ cycles/≤5% decay

## Data Availability

The original contributions presented in this study are included in the article. Further inquiries can be directed to the corresponding author.
